# Redetermination of diammonium thio­molybdate

**DOI:** 10.1107/S1600536810003016

**Published:** 2010-01-30

**Authors:** Björn Hill, Hans-Wolfram Lerner, Michael Bolte

**Affiliations:** a Institut für Anorganische Chemie der Goethe-Universität Frankfurt, Max-von-Laue-Strasse 7, D-60438 Frankfurt am Main, Germany

## Abstract

In contrast to the previous structure determinations of the title structure, (NH_4_)_2_[MoS_4_], the present determination at 173 K localized the positions of the H atoms. The title structure belongs to the β-K_2_SO_4_ family and all the ions are located on crystallographic mirror planes. The ions are held together by N—H⋯S hydrogen bonds (some of which are bifurcated), forming a three-dimensional network. One of the N atoms has nine contacts to the S atoms shorter than 4 Å, and the other has ten.

## Related literature

For preparation of the title compound, see: Herzog *et al.* (1981 ▶). For structures of the β-K_2_SO_4_ family, see: Fábry & Pérez-Mato (1994 ▶). For other structure determinations of the title compound, see: Lapasset *et al.* (1976 ▶); Schäfer *et al.* (1964 ▶). For a description of the Cambridge Structural Database, see: Allen (2002 ▶).

## Experimental

### 

#### Crystal data

(NH_4_)_2_[MoS_4_]
*M* *_r_* = 260.26
Orthorhombic, 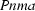

*a* = 9.5867 (4) Å
*b* = 6.9451 (4) Å
*c* = 12.2005 (5) Å
*V* = 812.32 (7) Å^3^
*Z* = 4
Mo *K*α radiation
μ = 2.55 mm^−1^
*T* = 173 K
0.25 × 0.24 × 0.11 mm

#### Data collection

Stoe IPDS II two-circle diffractometer
Absorption correction: multi-scan (*MULABS*; Spek, 2009 ▶; Blessing, 1995 ▶) *T*_min_ = 0.569, *T*_max_ = 0.767
14872 measured reflections
859 independent reflections
833 reflections with *I* > 2σ(*I*)
*R*_int_ = 0.072

#### Refinement

*R*[*F* ^2^ > 2σ(*F* ^2^)] = 0.022
*wR*(*F* ^2^) = 0.060
*S* = 1.19
859 reflections
61 parameters
6 restraints
H atoms treated by a mixture of independent and constrained refinement
Δρ_max_ = 0.51 e Å^−3^
Δρ_min_ = −0.88 e Å^−3^

### 

Data collection: *X-AREA* (Stoe & Cie, 2001 ▶); cell refinement: *X-AREA*; data reduction: *X-AREA*; program(s) used to solve structure: *SHELXS97* (Sheldrick, 2008 ▶); program(s) used to refine structure: *SHELXL97* (Sheldrick, 2008 ▶); molecular graphics: *XP* in *SHELXTL-Plus* (Sheldrick, 2008 ▶); software used to prepare material for publication: *SHELXL97* and *PLATON* (Spek, 2009 ▶).

## Supplementary Material

Crystal structure: contains datablocks I, global. DOI: 10.1107/S1600536810003016/fb2179sup1.cif

Structure factors: contains datablocks I. DOI: 10.1107/S1600536810003016/fb2179Isup2.hkl

Additional supplementary materials:  crystallographic information; 3D view; checkCIF report

## Figures and Tables

**Table 1:** Hydrogen-bond geometry (Å, °)

*D*—H⋯*A*	*D*—H	H⋯*A*	*D*⋯*A*	*D*—H⋯*A*
N1—H1*A*⋯S1^i^	0.86 (2)	2.76 (4)	3.491 (3)	144 (6)
N1—H1*B*⋯S1^ii^	0.88 (2)	2.92 (5)	3.607 (3)	135 (5)
N1—H1*B*⋯S3	0.88 (2)	2.87 (3)	3.497 (3)	129 (3)
N1—H1*B*⋯S3^iii^	0.88 (2)	2.87 (3)	3.497 (3)	129 (3)
N1—H1*C*⋯S3^iv^	0.88 (2)	2.76 (3)	3.550 (3)	150 (4)
N1—H1*C*⋯S3^iv^	0.88 (2)	2.76 (3)	3.550 (3)	150 (4)
N2—H2*C*⋯S3^v^	0.88 (2)	2.65 (3)	3.405 (2)	144 (4)
N2—H2*A*⋯S3^vi^	0.88 (2)	2.76 (2)	3.481 (2)	140 (1)
N2—H2*A*⋯S3^vii^	0.88 (2)	2.76 (2)	3.481 (2)	140 (1)
N2—H2*B*⋯S1	0.88 (2)	2.61 (4)	3.414 (3)	153 (6)
N2—H2*B*⋯S2	0.88 (2)	2.70 (6)	3.250 (3)	122 (5)

Symmetry codes: (i) 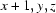; (ii) 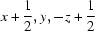; (iii) 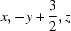; (iv) 
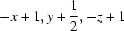
; (v) 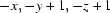; (vi) 
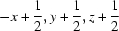
; (vii) 
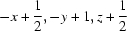
.

**Fig. 1.**
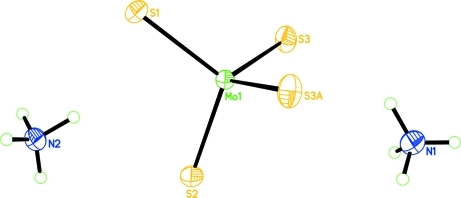
A view of the three molecules in the asymmetric unit of the title compound, with the atom-numbering scheme. The displacement ellipsoids are drawn at the 50% probability level and the H atoms are shown as small spheres of arbitrary radii.

## supplementary crystallographic information

### Comment

The crystal structure of the title compound, (NH_4_)_2_MoS_4_, previously determined using Weissenberg exposures (Schäfer *et al.*, 1964) and using a point detector diffractometer (Lapasset *et al.*, 1976) has been redetermined at low temperature since the two previous structure determinations did not include the positions of the H atoms.

The crystal structure belongs to the β-K_2_SO_4_ family (Fábry & Pérez-Mato, 1994). The anions and cations are held together by N—H···S hydrogen bonds forming a three-dimensional network involving all H atoms.

### Experimental

The ammonium tetrathiomolybdate (NH_4_)_2_MoS_4_ was synthesized by the reaction from (NH_4_)_6_Mo_7_O_24_^.^4H_2_O with H_2_S in the presence of NH_3_ (Herzog *et al.*, 1981) as shown by the equation:

(NH_4_)_6_Mo_7_O_24_ + 28 H_2_S + 8 NH_3_→ 7(NH_4_)_2_MoS_4_ + 24 H_2_O.

H_2_S was bubbled for 30 minutes through a solution of 4.94 g (4.0 mmol) (NH_4_)_6_Mo_7_O_24_^.^4H_2_O in 50 ml aqueous ammonia. At first the reaction solution became yellow then the colour changed from yellow towards red. The red colour indicated the end of the reaction (Herzog *et al.*, 1981). X-ray quality crystals of (NH_4_)_2_MoS_4_ were grown from the reaction solution at ambient temperature. The crystals are pleochroic, changing colour from red to green according to the view angle.

### Refinement

Hydrogen atoms were located in a difference Fourier map and refined isotropically. The N—H distances were restrained to 0.878 (20) Å. The value 0.878Å has been retrieved from the structures XUDGET, TERNOT, TEJMUQ, TEJMOK, KOLKAY, KEVVEN, ICOMUI contained in the Cambridge Crystallographic Database (Version 5.31; Allen, 2002). The condition of the search in the Cambridge Crystallographic Database: The structures contained [NH_4_]^+^, K was the possibly heaviest atom in the structure, and the structures have been determined with R-factor 〈 0.03.

### Crystal data

**Table:**

(NH_4_)_2_[MoS_4_]	*F*(000) = 512
*M**_r_* = 260.26	*D*_x_ = 2.128 Mg m^−^^3^
Orthorhombic, *P**n**m**a*	Mo *K*α radiation, λ = 0.71073 Å
Hall symbol: -P 2ac 2n	Cell parameters from 14093 reflections
*a* = 9.5867 (4) Å	θ = 2.7–26.4°
*b* = 6.9451 (4) Å	µ = 2.55 mm^−^^1^
*c* = 12.2005 (5) Å	*T* = 173 K
*V* = 812.32 (7) Å^3^	Plate, dark green
*Z* = 4	0.25 × 0.24 × 0.11 mm

### Data collection

**Table:**

Stoe IPDS II two-circle diffractometer	859 independent reflections
Radiation source: fine-focus sealed tube	833 reflections with *I* > 2σ(*I*)
graphite	*R*_int_ = 0.072
ω scans	θ_max_ = 25.9°, θ_min_ = 2.7°
Absorption correction: multi-scan (*MULABS*; Spek, 2009; Blessing, 1995)	*h* = −11→11
*T*_min_ = 0.569, *T*_max_ = 0.767	*k* = −8→8
14872 measured reflections	*l* = −14→15

### Refinement

**Table:**

Refinement on *F*^2^	Secondary atom site location: difference Fourier map
Least-squares matrix: full	Hydrogen site location: difference Fourier map
*R*[*F*^2^ > 2σ(*F*^2^)] = 0.022	H atoms treated by a mixture of independent and constrained refinement
*wR*(*F*^2^) = 0.060	*w* = 1/[σ^2^(*F*_o_^2^) + (0.0377*P*)^2^ + 0.2462*P*] where *P* = (*F*_o_^2^ + 2*F*_c_^2^)/3
*S* = 1.19	(Δ/σ)_max_ < 0.001
859 reflections	Δρ_max_ = 0.51 e Å^−^^3^
61 parameters	Δρ_min_ = −0.88 e Å^−^^3^
6 restraints	Extinction correction: *SHELXL97* (Sheldrick, 2008), Fc^*^=kFc[1+0.001xFc^2^λ^3^/sin(2θ)]^-1/4^
Primary atom site location: structure-invariant direct methods	Extinction coefficient: 0.0173 (13)

### Special details

**Table:**

Geometry. All e.s.d.'s (except the e.s.d. in the dihedral angle between two l.s. planes) are estimated using the full covariance matrix. The cell e.s.d.'s are taken into account individually in the estimation of e.s.d.'s in distances, angles and torsion angles; correlations between e.s.d.'s in cell parameters are only used when they are defined by crystal symmetry. An approximate (isotropic) treatment of cell e.s.d.'s is used for estimating e.s.d.'s involving l.s. planes.
Refinement. Refinement of *F*^2^ against ALL reflections. The weighted *R*-factor *wR* and goodness of fit *S* are based on *F*^2^, conventional *R*-factors *R* are based on *F*, with *F* set to zero for negative *F*^2^. The threshold expression of *F*^2^ > σ(*F*^2^) is used only for calculating *R*-factors(gt) *etc*. and is not relevant to the choice of reflections for refinement. *R*-factors based on *F*^2^ are statistically about twice as large as those based on *F*, and *R*- factors based on ALL data will be even larger.

### Fractional atomic coordinates and isotropic or equivalent isotropic displacement parameters (Å^2^)

**Table:**

	*x*	*y*	*z*	*U*_iso_*/*U*_eq_	
Mo1	0.25414 (2)	0.7500	0.42734 (2)	0.01490 (16)	
S1	0.03014 (7)	0.7500	0.38769 (6)	0.0229 (2)	
S2	0.28425 (9)	0.7500	0.60460 (7)	0.0246 (2)	
S3	0.35338 (5)	0.49616 (7)	0.35742 (5)	0.0259 (2)	
N1	0.6660 (3)	0.7500	0.3880 (2)	0.0257 (6)	
H1A	0.746 (4)	0.7500	0.355 (5)	0.072 (19)*	
H1B	0.590 (4)	0.7500	0.347 (4)	0.089 (19)*	
H1C	0.665 (5)	0.850 (5)	0.433 (3)	0.114 (18)*	
N2	−0.0471 (3)	0.7500	0.6609 (2)	0.0223 (5)	
H2A	−0.002 (5)	0.7500	0.723 (3)	0.09 (2)*	
H2B	0.003 (6)	0.7500	0.600 (3)	0.10 (2)*	
H2C	−0.097 (5)	0.644 (5)	0.656 (4)	0.114 (16)*	

### Atomic displacement parameters (Å^2^)

**Table:**

	*U*^11^	*U*^22^	*U*^33^	*U*^12^	*U*^13^	*U*^23^
Mo1	0.0146 (2)	0.0149 (2)	0.0152 (2)	0.000	0.00038 (8)	0.000
S1	0.0161 (4)	0.0297 (4)	0.0228 (4)	0.000	−0.0034 (3)	0.000
S2	0.0214 (4)	0.0349 (4)	0.0175 (4)	0.000	−0.0024 (3)	0.000
S3	0.0235 (3)	0.0196 (3)	0.0346 (3)	0.00079 (18)	0.0062 (2)	−0.0074 (2)
N1	0.0235 (14)	0.0268 (14)	0.0267 (15)	0.000	−0.0028 (11)	0.000
N2	0.0219 (13)	0.0258 (13)	0.0193 (13)	0.000	0.0030 (10)	0.000

### Geometric parameters (Å, °)

**Table:**

Mo1—S3^i^	2.1773 (5)	N1—H1B	0.88 (2)
Mo1—S3	2.1773 (5)	N1—H1C	0.883 (19)
Mo1—S2	2.1818 (9)	N2—H2A	0.88 (2)
Mo1—S1	2.2013 (8)	N2—H2B	0.88 (2)
N1—H1A	0.86 (2)	N2—H2C	0.879 (19)
			
S3^i^—Mo1—S3	108.13 (3)	H1A—N1—H1B	118 (6)
S3^i^—Mo1—S2	109.30 (2)	H1A—N1—H1C	107 (4)
S3—Mo1—S2	109.30 (2)	H1B—N1—H1C	110 (3)
S3^i^—Mo1—S1	109.885 (19)	H2A—N2—H2B	117 (5)
S3—Mo1—S1	109.885 (19)	H2A—N2—H2C	109 (3)
S2—Mo1—S1	110.30 (3)	H2B—N2—H2C	103 (3)

Symmetry codes: (i) *x*, −*y*+3/2, *z*.

### Hydrogen-bond geometry (Å, °)

**Table:**

*D*—H···*A*	*D*—H	H···*A*	*D*···*A*	*D*—H···*A*
N1—H1A···S1^ii^	0.86 (2)	2.76 (4)	3.491 (3)	144 (6)
N1—H1B···S1^iii^	0.88 (2)	2.92 (5)	3.607 (3)	135 (5)
N1—H1B···S3	0.88 (2)	2.87 (3)	3.497 (3)	129 (3)
N1—H1B···S3^i^	0.88 (2)	2.87 (3)	3.497 (3)	129 (3)
N1—H1C···S3^iv^	0.88 (2)	2.76 (3)	3.550 (3)	150 (4)
N1—H1C···S3^iv^	0.88 (2)	2.76 (3)	3.550 (3)	150 (4)
N2—H2C···S3^v^	0.88 (2)	2.65 (3)	3.405 (2)	144 (4)
N2—H2A···S3^vi^	0.88 (2)	2.76 (2)	3.481 (2)	140 (1)
N2—H2A···S3^vii^	0.88 (2)	2.76 (2)	3.481 (2)	140 (1)
N2—H2B···S1	0.88 (2)	2.61 (4)	3.414 (3)	153 (6)
N2—H2B···S2	0.88 (2)	2.70 (6)	3.250 (3)	122 (5)

Symmetry codes: (ii) *x*+1, *y*, *z*; (iii) *x*+1/2, *y*, −*z*+1/2; (i) *x*, −*y*+3/2, *z*; (iv) −*x*+1, *y*+1/2, −*z*+1; (v) −*x*, −*y*+1, −*z*+1; (vi) −*x*+1/2, *y*+1/2, *z*+1/2; (vii) −*x*+1/2, −*y*+1, *z*+1/2.
